# A novel substitution matrix fitted to the compositional bias in Mollicutes improves the prediction of homologous relationships

**DOI:** 10.1186/1471-2105-12-457

**Published:** 2011-11-24

**Authors:** Claire Lemaitre, Aurélien Barré, Christine Citti, Florence Tardy, François Thiaucourt, Pascal Sirand-Pugnet, Patricia Thébault

**Affiliations:** 1 Université de Bordeaux, Centre de Bioinformatique et Génomique Fonctionnelle Bordeaux, F-33000 Bordeaux, France; 2 Equipe SYMBIOSE, INRIA Rennes Bretagne Atlantique, Campus de Beaulieu, F-35042 Rennes, France; 3 Université de Toulouse, ENVT, UMR 1225, F-31076 Toulouse, France; 4 INRA, UMR 1225, F-31076 Toulouse, France; 5 Anses, Lyon Laboratory, UMR Mycoplasmoses of Ruminants, 31 Avenue Tony Garnier F-69364 Lyon cedex 07, France; 6 CIRAD, UMR CMAEE, Campus de Baillarguet, F-34398 Montpellier, France; 7 Université de Bordeaux, UMR 1332, 71, avenue Edouard Bourlaux, F-33140 Villenave d'Ornon, France; 8 INRA, UMR 1332, 71, avenue Edouard Bourlaux, F-33140 Villenave d'Ornon, France; 9 Université de Bordeaux, Laboratoire Bordelais de Recherche en Informatique, UMR 5800, F-33405 Talence, France

## Abstract

**Background:**

Substitution matrices are key parameters for the alignment of two protein sequences, and consequently for most comparative genomics studies. The composition of biological sequences can vary importantly between species and groups of species, and classical matrices such as those in the BLOSUM series fail to accurately estimate alignment scores and statistical significance with sequences sharing marked compositional biases.

**Results:**

We present a general and simple methodology to build matrices that are especially fitted to the compositional bias of proteins. Our approach is inspired from the one used to build the BLOSUM matrices and is based on learning substitution and amino acid frequencies on real sequences with the corresponding compositional bias. We applied it to the large scale comparison of Mollicute AT-rich genomes. The new matrix, MOLLI60, was used to predict pairwise orthology relationships, as well as homolog families among 24 Mollicute genomes. We show that this new matrix enables to better discriminate between true and false orthologs and improves the clustering of homologous proteins, with respect to the use of the classical matrix BLOSUM62.

**Conclusions:**

We show in this paper that well-fitted matrices can improve the predictions of orthologous and homologous relationships among proteins with a similar compositional bias. With the ever-increasing number of sequenced genomes, our approach could prove valuable in numerous comparative studies focusing on atypical genomes.

## Background

A fundamental task in evolutionary biology and comparative genomics is to quantify the similarity between biological sequences and then assess their evolutionary relationships. The challenge is in particular to distinguish between homologous (and even orthologous and paralogous) and unrelated sequences. DNA and protein sequences similarities are usually assessed through an alignment of sequences. The scoring function is crucial in the alignment process: not only it enables to choose the best pairing between the sequences among all possible, but also it is often at the heart of the evaluation of the alignment significance. Indeed, a p-value or e-value is usually derived from the score of the alignment to assess if it is statistically different from one that can arise by chance (in a random alignment). Substitution matrices are therefore key parameters in the alignment process since they assign an elementary score to each match and mismatch between any two letters of the alphabet. This is particularly relevant in the case of protein alignment. As the protein alphabet contains 20 amino acids with different frequencies in biological sequences and different biochemical properties that may impact the protein structure, matches and mismatches between amino acids are not equipropable. It is thus crucial to take into account these differences in order to estimate accurately the similarity between sequences.

The most widely used matrices are the BLOSUM [1] and PAM [2] series. They were both derived from real sequences where the substitution frequencies between amino acids were observed. The two approaches differ in the way they derive several matrices, each of them reflecting a given level of sequence divergence. Whereas the PAM approach uses a model of evolution to derive new matrices at a given amount of evolutionary time starting from an inferred matrix scaled for a unit of time, the BLOSUM one is purely empirical and infer substitution frequencies for each matrix from observations on sequences with the given level of divergence.

These matrices are typically expressed in log-odds scores: the score of the pairing of two amino acids is the logarithm of the ratio of the likelihoods of this pairing under two hypotheses: homology versus chance. It reflects thus how likely two aligned residues are descendants of a common ancestral residue with respect to random pairing: if the pairing of two residues is more likely to arise by chance than by homology the score will be negative, in the opposite situation it will be positive. Consequently, two probabilities need to be computed: the one of observing the two residues aligned in an alignment of homologous sequences and the probability of observing the two residues aligned in unrelated sequences. The latter can be easily computed when assuming independence between the sequences, that is the probability of drawing at random these two residues among all residues present in the sequences, it is thus the product of their frequencies in the sequences, also called the background frequencies. The first probability, also called the target frequency, is inferred by counting the amount of each kind of substitutions in homologous sequence alignments.

For both approaches, the reference set of homologous sequences used to estimate the substitution frequencies is therefore essential and the resulting matrices will reflect both the composition and the evolutionary characteristics of these sequences. For both matrix series, this reference set was composed of "classical" sequences and together with the large size of the datasets, this gave matrices with an "average" amino acid composition. However, sequence compositions can vary drastically, and one can find groups of sequences with atypical compositions: certain types of proteins have a composition directly correlated to their function, such as transmembrane proteins which include tracts of hydrophobic residues. Additionally, at the scale of the whole genome, some species show extreme nucleotide compositions that affect their amino acid composition, one of the most extreme example being the malaria agent, *Plasmodium falciparum*, with a 80%- A+T genome [3].

Keeping the rationale of log-odds scores in mind, we can easily figure out that the classical matrices will not perfectly fit sequences with biased amino acid composition. Basically, the likelihood of each match and mismatch under the null hypothesis of unrelated sequences will vary according to the differences in the background frequencies. For instance, for amino acids which are rare in classical sequences but much more abundant in the sequences of interest, the probability of observing their pairing just by chance would be greatly underestimated in the classical matrices and therefore their log-odds score will not reflect a "true" likelyhood ratio. As a consequence of these discrepancies the resulting alignment can be wrong (not pairing together homologous residues) and also mis-evaluated, both leading possibly to mis-interpretation of the similarity. This issue has already been pointed out from a theoretical point of view by Yu *et al. *[4] and several studies have highlighted the negative impacts of using classical matrices in homology and database searches in atypical compositional contexts [5-7]. These papers proposed several methods to modify the matrices and derive new ones fitted to some compositional biases. Two different approaches can be noticed. The first one is called matrix adjustment and consists in transforming the classical matrices by applying some mathematical formulas on its elements, taking into account the old and new amino acids background frequencies. The first adjustment was proposed by Yu *et al. *and further implemented in the well-known (and used) program BLAST [4,8]. Later, Coronado *et al. *deviced a similar method for low complexity sequences and showed an improvement over classical masking strategies [5]. The main advantage of these methods is their speed and usability on any sequence. However, their main drawback is that they only take into account the differences in the background frequencies, assuming the target frequencies are the same. Therefore they do not reflect specifities of mutation or selection biases in the substitutions, still leading to inaccurate likelihood ratio. Moreover the composition is estimated independently for each aligned sequence and consequently may not reflect the overall composition of the genome or group of proteins of interest. Since each protein is aligned with a different scoring function, comparisons of scores are not straightforward and may not be pertinent in the case of genome-scale comparative analyses. The second approach addresses these issues, and consists in building new matrices from scratch in a similar way to the BLOSUM or PAM approaches but using an initial dataset of sequences with the desired compositional bias. In this manner, two groups proposed simultaneously two similar methods based on the BLOSUM approach in order to compare the proteins of the *Plasmodium *genus [6,7]. Bastien *et al. *also used this approach to build asymetric matrices enabling to compare proteins with an atypical amino acid composition (again *Plasmodium *proteins) against "classical" proteins (*Arabidopsis thaliana*) [9]. These previous works have shown the feasibility and the benefits of such approaches, at least in the extreme case of Plasmodium. However these have not been shown for less extreme cases of compositional bias, nor for the comparison of a large number of genomes at diverse levels of divergence.

In this paper, we address the problem of designing new substitution matrices fitted to the compositional bias of Mollicute proteins. Mollicutes are a particular class of bacteria derived from Gram-positive bacteria, including for instance the well-known *Mycoplasma genitalium *species. Mollicutes share several atypical features such as a small cell size, the absence of a cell wall, a dramatically reduced genome with simplified metabolic pathways, and of particular interest here, a nucleotide composition biased toward A and T (median value of 27.1% G+C). They exhibit a wide variety of phenotypes with diverse host environments ranging from plants, arthropods to vertebrates, and also with various pathogenic or non-pathogenic impacts [10]. With the advent of new sequencing technologies, the number of Mollicute genomes fully sequenced has greatly increased in the last couple of years, making the comparative genomics analyses more challenging. Currently 38 whole genomes from 30 distinct species are publicly available in the reference database Molligen (http://cbib1.cbib.u-bordeaux2.fr/molligen3b/), some of which diverged up to 470 million years ago [11]. This constitutes therefore a large set of genomes with a similar compositional bias, and with high comparative interests at various evolutionary scales. The objective of this work is to increase the sensibility and specificity of homologous and orthologous predictions among this large number of genomes. We present in this paper the methodology developped to build a new matrix, MOLLI60, specially fitted to Mollicute proteins, and we show that it enables better performance in predicting orthologs and homologs with respect to the use of the classical matrix BLOSUM62. Finally, we argue that the methodology could be easily generalized to other cases of compositional bias and could be useful in numerous comparative studies, given the recent and massive increase in genomic resources.

## Results

### Compositional bias of Mollicute proteins

**Figure 1:**
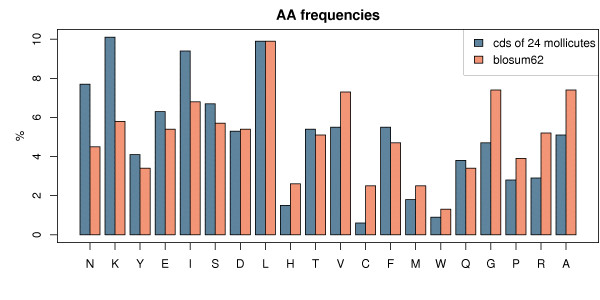
**Amino acid frequencies**. Amino acid frequencies averaged over all cds of 24 whole genomes of Mollicutes versus BLOSUM62's. A complete list of genomes taken into account is provided in Additional file 4.

The nucleotide composition of Mollicute genomes is strongly biased towards A and T, with a G+C content that varies from 21.4 to 40.0% with a median value of 27.1%. *Mycoplasma pneumoniae *appears as an exception among the Mollicutes, with a G+C content almost "normal" of 40%, wheras all the other sequenced Mollicutes have their G+C content less than 32%. As a consequence, the composition of their protein sequences is also biased towards certain amino acids. Strong differences between frequencies can be observed in Figure 1, where the average amino acid frequencies of the coding sequences of 24 Mollicute genomes are plotted against the ones derived from the usual BLOSUM62 matrix. For instance Isoleucine, Asparagine and Lysine are more numerous in Mollicute proteins, whereas Alanine, Arginine and Glycine are depleted. As expected the former are encoded by AT-rich codons, and the latter by GC-rich codons, suggesting that these frequency differences stem from the genomic nucleotide compositional bias. We confirmed this relationship by performing the so-called GARP-vs-FYMINK analysis [12] where we can observe, in mollicute coding sequences, a strong positive correlation between the proportion of GARP amino acids (encoded by GC-rich codons) and the GC content, and conversely a negative correlation with FYMINK amino acids (encoded by AT-rich codons) (data not shown). Given such differences in amino acid frequencies, we hypothesized that BLOSUM62 may not be well fitted for the purpose of aligning Mollicute protein sequences.

### MOLLI60: the new substitution matrix

The main result of this paper is the generation of a new substitution matrix, called MOLLI60, which is better fitted to the compositional bias of Mollicute protein sequences. We generated this matrix with an approach similar to the one used to generate the well-known BLOSUM matrices [1]. This consists in learning the amino acid substitution frequencies from the alignments of real sequences. Whereas the BLOSUM matrices were built from sequences originated from a variety of organisms, we selected here sequences only from Mollicutes to get frequencies representative of the compositional bias of these genomes. A curated set of 247 protein families from 14 mycoplasma genomes was used as learning set (see Methods). Sequences in each family were aligned with T-Coffee [13] and from these multiple alignments conserved gap-free blocks were extracted. This gave a set of 1510 blocks containing overall 64143 columns and 880058 total amino acids. This is the same order of magnitude of the the amount of data used to build the BLOSUM matrices, which was constituted of 2106 blocks and a total of 927076 amino acids. To estimate the substitution frequencies, highly similar sequences were clustered to obtain a matrix reflecting to a certain amount of divergence between the sequences to align. Thus, to compare with BLOSUM62 we used a similar clustering coefficient of 60 and scaled the matrix similarly as BLOSUM62 to obtain half-bit scores (see Methods). The matrix, MOLLI60, and the scripts to compute it are provided as supplementary material (Additional files 1 and 2).

Global features of the matrix were then investigated, such as the entropy and expected values. These are commonly computed for substitution matrices and reflect the level of stringency of the matrix. Typically, a matrix with a lower entropy and a greater expected value will reflect more diverged sequences. We reported also, in table 1, the average scores for mismatches and matches. Overall, we obtained very similar values between MOLLI60 and BLOSUM62, as compared with BLOSUM45 (see table 1), suggesting that both matrices have the same global properties.

**Table 1:** Comparison of entropy and expected values for several matrices

matrix	entropy	expected	mean mismatch	mean match
MOLLI60	0.7126	-0.5820	-1.56	6.10

BLOSUM62	0.6979	-0.5209	-1.42	5.80

BLOSUM45	0.3795	-0.2789	-1.34	7.05

**Figure 2:**
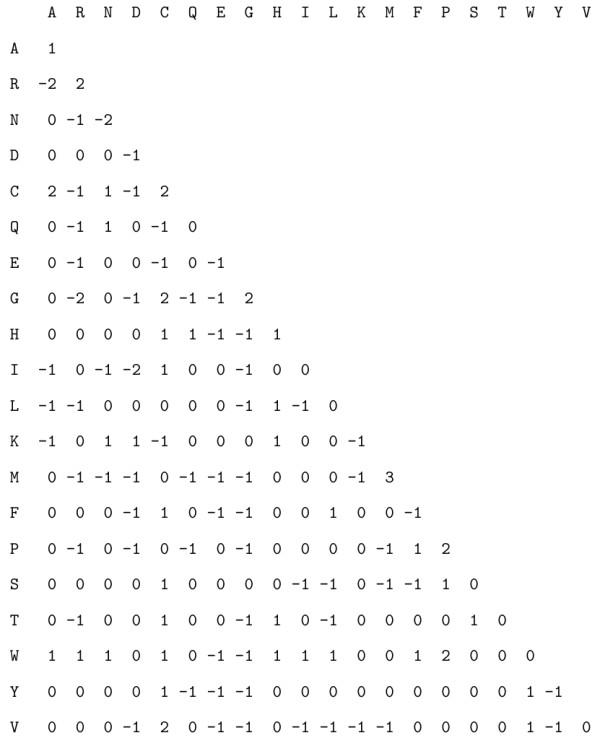
**matrix substraction: MOLLI60 - BLOSUM62**. Term by term substraction of the two matrices MOLLI60 minus BLOSUM62

However, when looking more closely at each individual element, we can see in Figure 2 that the two matrices are quite different with almost half the positions with different values (105 over 210 elements in the half matrix). Some substitutions show high score differences and logically the greatest differences are observed for amino acids with the greatest overall frequency differences (Figure 1), such as Arginine and Glycine. We can notice that for these two residues, which are under-represented in Mollicute proteins, their match score is increased (since it is less likely to observe them in an alignment of random sequences) whereas their mismatch scores are globally decreased, indicating their depletion also in substitutions in Mollicute proteins. An interesting difference is observed for the Tryptophan (W): whereas its frequencies are very close between Mollicutes and Blosum (Figure 1), this amino acid shows strong differences in the new matrix. This can be explained by a specificity of the genetic code of Mollicutes: they can encode Tryptophan with the additional codon UGA, which is usually a stop codon. Therefore MOLLI60 can take into account differences not only in the background frequencies, but also in the evolutionary dynamics of the substitutions.

The robustness of MOLLI60 with respect to the learning set composition was evaluated, using a re-sampling approach (see Methods). If we removed randomly 10% of the initial mutliple alignments, the resulting matrix was slightly modified with 3 to 8% of the positions with different values and when removing half of the data only 15% of the matrix positions were changed. Noteworthy, these changes are minor compared to the differences with BLOSUM62.

Since the initial alignments used to infer the substitutions were obtained using BLOSUM62 (not fitted to the bias), the impact of this first step was evaluated by iterating the matrix construction with MOLLI60. Actually, this recursive step appeared not necessary since the resulting matrix was very similar to the first one, showing only 10 elements over 210 with a -1 or +1 difference (less than 5% of the elements). The amount of differences is thus similar to the one obtained when removing 10% of the data.

### Application of MOLLI60 in homology predictions

We used the matrix MOLLI60 to align Mollicute protein sequences and to predict homology and orthology relationships based on these alignments.

#### Orthology predictions

The standard method of bi-directional best hit (BDBH) was used to predict orthologous genes between two genomes and proteic sequence alignments were obtained using the program SSEARCH, using either MOLLI60 or the standard BLOSUM62 as substitution matrix (see Methods).

We focused on 3 mycoplasma genomes for which we dispose of a reference set of orthologous relationships: *Mycoplasma genitalium*, *Mycoplasma hominis *and *Ureaplasma parvum*. This reference set contains 871 pairwise relationships which were manually curated by biologists (see Methods). It enables to evaluate orthology predictions and to compare the influences of different substitution matrices, by classifying the predictions as true positive, false positive or false negative.

When comparing orthologous predictions obtained using the standard BLOSUM62 matrix versus our matrix MOLLI60, we first noticed that alignments obtained with MOLLI60 have overall smaller e-values than the ones obtained with BLOSUM62: the median e-value of BDBH proteins obtained with BLOSUM62 is 5.9e-22 against 1.1e-31 for MOLLI60. If we consider only common pairwise relationships between the two sets, the median pairwise ratio of e-values is of 10^11 ^in favor of the alignments obtained with MOLLI60: half of the common alignments have e-values obtained with BLOSUM62 more than 10^11 ^larger than the ones obtained with MOLLI60. However, having better e-values does not necessarily mean that the alignments are better and enable to infer better biological information. What is important is to get scores or e-values that improve the discrimination between biologically meaningfull similarity and random similarity. If we consider now all the alignments obtained between all proteins (with still an e-value cut-off of 10), meaning that this set contains surely a large portion of non-homologous relationships (or poor similarity), we still get better e-values with the MOLLI60 alignments than the ones with BLOSUM62. However, the difference is significantly smaller than previously with a median ratio of only 5 (instead of 10^11^). Thus this suggests that the observed differences in e-values between the two matrices are not systematic and depends on the significance of the alignments.

**Figure 3:**
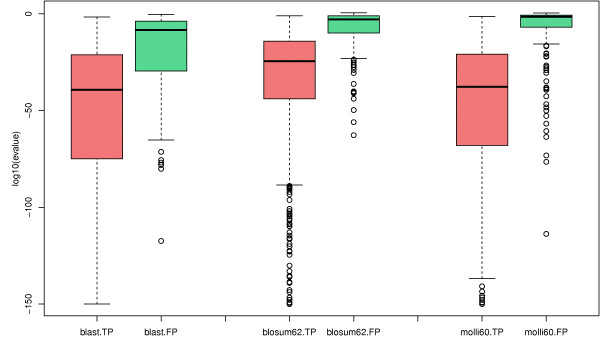
**Evalue discrimination**. Comparison of the distribitution of BDBH e-values between True Positive (TP in red) and False Positive (FP in green). On the left alignments were obtained with BLAST and the BLOSUM62 matrix, on the middle with SSEARCH and the BLOSUM62 matrix, and on the right with SSEARCH and the MOLLI60 matrix.

To evaluate the discriminative power of the e-values, we plotted in Figure 3 the distributions of e-values between true positive and false positive predictions obtained by BDBH. We can clearly see that the alignments obtained with MOLLI60 show a distribution with more disciminated e-values between these two categories compared to BLOSUM62. Indeed, the median e-value of MOLLI60 true positive alignments is smaller than the BLOSUM62's one (10^-38 ^against 10^-25^) and it is the contrary for false positive alignments (0.026 against 0.00125). Overall the mean ratio of e-values between false and true positives is of 10^45 ^for MOLLI60 against 10^25 ^for BLOSUM62, thus the discrimination between true and false positive e-values is significantly improved when using MOLLI60 (p-value of 4.96*e *- 05, test of Analysis of Variance, see the Methods section).

**Figure 4:**
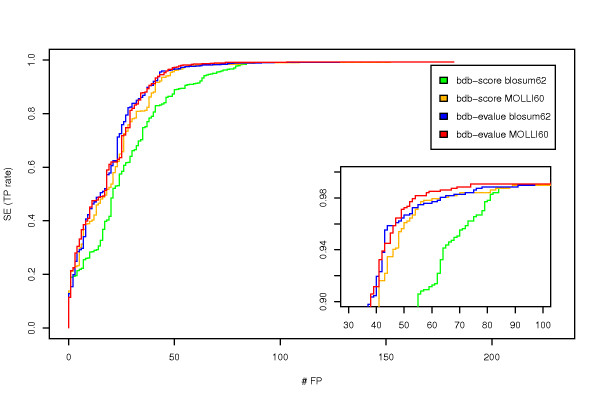
**ROC curves**. ROC curves of one-to-one orthologous relationship predictions using the Bi-directional Best Hit method with several best-hit criteria and substitution matrices. Either the score (bdb-score) or the e-value (bdb-evalue) was used to determine the best hit, and the alignments were obtained with SSEARCH using either the BLOSUM62 matrix or the MOLLI60 matrix. As inset, is a zoom-in on the most interesting part of the curve: where the trade-off between sensitivity and specifity is usually determined for orthology predictions. Note that sensitivity (or true positive rate) is plotted against the absolute number of false positives instead of the false positive rate as in a classical ROC curve, for lisibility reasons. FP rate can be obtained simply by dividing the amount of FP by the amount of non-orthologous relationships which is constant (884833).

For both matrices, we computed also their sensitivity and specificity (see Methods). As the two matrices give different e-values even for the same alignment, it is not pertinent to compare their sensitivity and specificity when using a fixed e-value cut-off to predict orthologs. Therefore we represented them using the Receiver Operating Characteristic (ROC) curves [14]. This type of curve enables to visualise how sensitivity and specificity simultaneously vary over the whole range of cut-off values. When plotting the sensitivity as a function of the false positive rate, the best method is assessed as the one whose curve is closest to the top-left corner (high sensitivity and low false positive rate). We plotted ROC curves in Figure 4 for the BDBH obtained with the two matrices. We can see that MOLLI60 and BLOSUM62 have very similar curves with the MOLLI60 one slighlty "over" the BLOSUM62. If the difference between ROC curves is not statistically significant when using evalues to call the best-hits (comparing the area under the curves, p-value of 0.9632), the difference is greater and significant when using raw scores (p-value of 0.001). This indicates that BLOSUM62 scores are less meaningfull than MOLLI60 ones. Additionally, we can also notice that, regardless of the matrix, the raw score is less meaningfull than the e-value for the purpose of predicting orthologous relationships. Finally, the same analysis was performed using the program BLAST and showed that using the same substitution matrix, BLOSUM62, the strategy with SSEARCH performs better than BLAST to predict BDBH orthologs, even when correcting for the compositional bias with BLAST -C3 option (Additional File 3, Figure S1). In particular, we observed that the adjustment process improves the specifity of the predictions but with the cost of an important loss in sensitivity: without any filtering of the BDBH pairs (that is when relying only on the reciprocal best hit criterion), 22 and 12 relationships are definitely lost with BLAST using repectively -C3 and default parameter compared to at most 8 with SSEARCH. This may be due to the gap penalties no longer adapted to the adjusted matrix since we observed that alignments were on average 10% smaller with the adjustment option.

Importantly, we verified that these results were not due to the fact that the training dataset used to build the matrix had common sequences with the test dataset. To do so, we built a second matrix without using any sequence of the genomes of the test dataset (*Mycoplasma genitalium*, *Mycoplasma hominis *and *Ureaplasma parvum*) and re-did these analyses. We obtained a very similar matrix and same results of comparison with BLOSUM62 on BDBH relationships (data not shown).

#### Homologous family predictions

We applied our matrix also to predict homologous protein families among 24 Mollicute genomes. The prediction of homologous relationships is more challenging that predicting orthologous ones (and especially one-to-one relationships), since we can not rely anymore on the best hit criterion. Instead, we have to use a cut-off to decide if two proteins are homologous and a clustering approach to build groups where each member is assumed to be homologous to any other member of the group. The construction of homologous clusters was performed using a naive approach based on an e-value cut-off: any two proteins with their alignment e-value lower than the cut-off are grouped in the same cluster (this corresponds to single-linkage clustering, see the Methods section). This implies that two proteins can belong to the same cluster even if they do not share enough similarity, for instance they only need to be both related to a third protein. This transitivity property is justified by the definition of homology of a common ancestor, which implies that if a and b are homologous and so are a and c, then b and c are also homologous.

Considering such process, and as the best hit criterion is no more usefull, we guess that the impact of the substitution matrix on these predictions may be more significant than for BDBH predictions. However in the absence of a reference dataset, the estimation of sensitivity and specificity scores is not applicable. Instead, we can compare the number of clusters, their size and their phylogenetic profile (presence/absence of species). We call the pan genome the number of distinct clusters (including also the single proteins) among the set of genomes, and the core genome the number of distinct clusters containing at least one member of each species. Assuming that each cluster represents a distinct funtion, the pan genome may be seen as the set of functions one can find in the community of genomes and the core genome the set of functions shared by all genomes. The number and size of clusters depend naturally on the e-value threshold: the more stringent is the threshold the more numerous and the smaller the clusters are, in other words the larger both the pan and the core genomes are. We can expect that a good clustering would give the fewest singletons (that is single proteins without any homolog), and consequently the smallest pan genome. On the other hand, a clustering with too many large clusters may group together several distinct families and thus reduce the core genome. Therefore when comparing several clustering we should prefer the one with the largest core genome and the smallest pan genome.

**Figure 5:**
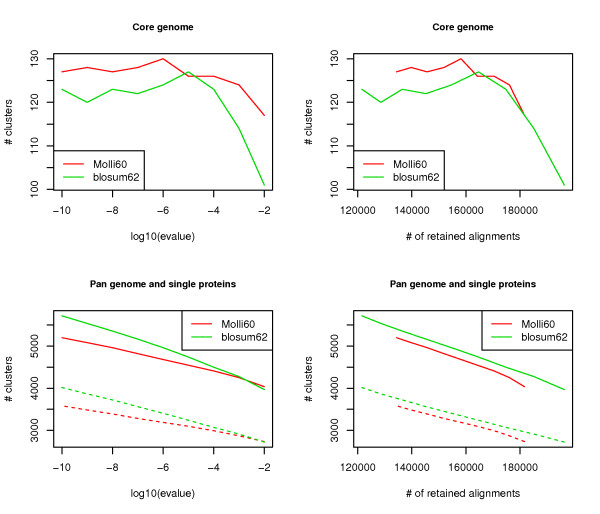
**Core and pan genomes**. Comparison of core (top) and pan (bottom) genome sizes for clustering obtained with the two matrices, as a function of the e-value threshold (left) or the number of retained alignments (right). The dash lines represent the number of unclustered proteins, ie. single proteins.

Since the core and pan genomes depend on the e-value threshold, and we have seen previously that e-values are not directly comparable between the two matrices, we built several clustering for each matrix by varying the e-value cut-off and computed for each their core and pan genomes. The results are presented in Figure 5 where the sizes of the core and pan genomes are plotted as a function of the threshold, or alternatively of the number of pairwise alignments retained with a given cut-off. We can clearly see that for the same number of pairwise alignments, the clustering obtained with MOLLI60 gives at the same time a larger core genome and a smaller pan one than the clustering obtained with BLOSUM62. Thus the clustering with MOLLI60 is better on both fronts and seems to give more pertinent clusters from a parsimonious evolutionary standpoint (see Discussion).

As a complementary view, we evaluated the homogeneity of the families in terms of domain compositions of their protein members. Assuming domain architecture is conserved inside families, we expect that a good clustering will have a common domain architecture inside each family and different ones between families. For 14% of the proteins we could predict a domain architecture based on the Pfam-A database (see the Methods section). For a given clustering, we computed two metrics of domain homogeneity: *intra *- *discrepancy *as the number of families with more than one domain architecture weighted by the number of different architectures, and *inter *- *discrepancy *as the number of pairs of families sharing at least one domain architecture. We compared these metrics for the clusterings obtained with MOLLI60 versus BLOSUM62 giving the largest core genomes (thresholds of evalue of 1e-6 and 1e-5 respectively). For both metrics the clustering obtained with MOLLI60 showed a better agreement with domain predictions: its *intra *- *discrepancy *value being of 129 versus 139 for BLOSUM62, and 1302 for *inter *- *discrepancy *versus 1465. This result holds also for other evalue thresholds (Additional File 3, Figure S2).

Based on these results, the matrix MOLLI60 was applied for the generation of comparative data in the reference database Molligen (predictions of orthologous and homologous relationships) and it is proposed as an optional parameter for the alignment tools provided to query the database.

## Discussion

We want to emphasize that, although our results are focused on a specific group of species, the Mollicutes, the method for building the matrix and the analyses of its impacts can be easily generalized to any group of species with an atypical composition.

### Methodology to build the matrix

To build this new matrix, we followed the approach initially proposed by Henikoff and Henikoff to build the BLOSUM series [1]. The main difference lies in the building of the block database. Henikoff and Henikoff used the motif finder PROTOMAT [15] to avoid relying on an initial substitution matrix. Instead we used a multiple alignment program and, as dicussed below, we showed that the initial matrix used for the alignments of the block database had little impact on the resulting matrix. Concerning the more recent methods developped especially for *Plasmodium falciparum*, our method differs mainly with respect to the learning sets of proteins. The sets of Bastien *et al *and Paila *et al*, included few sequences from genomes with the bias and more sequences with no bias, in particular to build non-symetrical matrices [7,9], and in Brick *et al *the learning set was not composed of sequences from the genomes of interest but by selecting blocks from the BLOSUM database showing a similar compositional bias. Having at our disposal a large set of genomes sharing the same compositional bias, we could stick to the BLOSUM strategy and take full advantage of it.

As for the global approach, we chose to follow the BLOSUM strategy rather than the PAM one mainly because it has been shown to perform better in homology search context [16]. Furthermore the substitution frequencies are learned directly from the data and are not extrapolated with an evolutionary model which may not reflect the evolution of our proteins. Additionally, the method has few parameters to adjust, with an intuitive biological meaning (e.g minimal size of a conserved block, clustering coefficient as an identity percentage). More importantly, the major drawback of the PAM strategy is the restriction on the input sequences: it requires highly similar sequences (typically with more than 85% of identity) which would have considerably reduced the size of our learning set. This is clearly a limiting factor when dealing with a small number of genomes or non model organisms with a limited reference data set.

Actually, the major hurdle of our strategy is its reliance on a reference set of orthologous proteins, as opposed to methods that only adjust the standard matrices to the bias with theoritical formulas [4,5]. One needs thus to have already a certain expertise on the genomes of interest. Moreover, this set has to be representative of all the genomes and proteins one wants to apply the matrix on. In our case, we used orthologous proteins conserved among 14 Mollicute genomes, for which there was no ambiguity in their orthologous relationships. We assumed that the wide distribution of these species in the phylogenetic tree of Mollicutes, along with the protein set covering diverse biological functions (as a large part of the core genome), prevented our model to be over-fitted to a specific genome or protein family. Note that since these proteins are probably shared among all or most Mollicutes, they may evolve more slowly than the remaining proteins. However, even if the substitutions are expected to be less frequent, we assumed that the relative rates between the different amino acids are representative of Mollicute protein evolution, and thus this sampling bias would have little impact on the matrix.

As concerns this learning dataset, we also verified that the final matrix was not too dependant on some individual parts of this set, by a re-sampling approach. This suggested that our learning set was homogeneous enough to estimate correct substitution rates. We also noticed that a recursive step where multiple alignments would be obtained with the new matrix instead of the standard BLOSUM62 had few impacts on the resulting matrix. This suggests that substitution matrices may be less important in multiple alignment than pairwise alignment and especially for well conserved proteins. Indeed, the signal of conservation, if it exists, is easier to detect when we deal not only with two sequences but with a large set of sequences. Moreover, we used a multiple aligner, T-Coffee, which is based on consistency scores rather than substitution scores and may thus be less sensitive to differences in substitution matrices. Additionally, in multiple alignments, we align sequences already known as homologous and we do not need nor use the scoring of the alignment. In the context of pairwise alignment for homology search, substitution matrices may have more impact on the evaluation of the alignment rather than on the alignment itself (ie. which position is aligned against which one).

Finally, the last but non negligible step of the method is the optimisation of the gap parameters to the new matrix. In gapped alignments, gap penalties have a strong impact on the alignments and especially their length. Indeed, the score of a gap has to be fixed relatively to the scores of matches and mismatches. The task of adjusting the gap penalties to the matrix is not trivial and there exists no theoretical formula to relate them to some features of the matrix [17]. We thus followed an empirical approach, testing numerous combinations of gap penalties and choosing the one maximizing the performances of BDBH predictions. Notably, we observed that small changes in the parameters of the matrix construction, such as the clustering coefficient and the normalisation parameter λ, could change dramatically the optimal gap penalties. This approach turned out to give the same penalties for both matrices, and this may be related to the similar entropy and expected values of both matrices. Nevertheless we want to stress out here on the importance of parameterisation of the gap penalties in pairwise alignments and database searches.

### Evaluation of the matrix

Pairwise alignments and similarity searches in databases are the basis of numerous comparative genomics analyses. What is important in this step is to be able to discriminate between biologically meaningfull similarities and random ones. We showed in this paper that this can be improved by using well fitted substitution matrices. We demonstrated that MOLLI60 enabled to better evaluate pairwise alignments with raw-score, as well as e-values. The difference with BLOSUM62 was particularly significant for the raw score (Figure 4), enhancing the matrix influence since it is directly derived from it. The difference was smaller for e-values and this suggests that the mis-evaluation of the scores can be compensated by the statistical analysis of their distribution which is performed by SSEARCH when estimating e-values. Nevertheless, we could still see an effect with e-values especially in homology clustering.

In this paper, we also compared two alignment programs: SSEARCH [18] and BLAST [19]. We showed that SSEARCH performs better to predict one-to-one orthologs than BLAST with the same parameters, at least in this context of compositional bias. Noteworthy, SSEARCH still outperforms BLAST with its compositional adjustment option. This result is to relate to two major differences between the programs. First, SSEARCH performs exact local alignments (Smith and Waterman algorithm) and thus garantees to find the best scoring alignment between two sequences, contrary to BLAST which is a heuristic. Note that, even if Smith and Waterman algorithm has worse theoretical time and spatial complexities (*O *(*n*^2^)), its implementation in SSEARCH as multi-threaded and vectorized greatly improves the runtime, which is no longer a limiting factor. Secondly, they differ in the way they evaluate alignments. BLAST computes e-values using a formula with pre-computed statistical parameters [20], whereas SSEARCH estimates the e-value of a given alignment using the distribution of scores obtained from aligning the same query sequence against all the sequences in the database. Therefore, SSEARCH's approach does not rely on simulations with artificial sequences to estimate parameters, as in BLAST, and it takes fully into account the compositional bias of the database and the query sequences. These two differences also guided us to favor SSEARCH for the comparison of the matrices, since they ensure that the observed performance differences are only due to the matrices and not partly linked to the heuristic approach or the simulations performed to estimate the statistical parameters which could also depend on the matrix. This is also the reason why we chose simple methods with few parameters to predict orthologous and homologous relationships, such as BDBH and single linkage clustering, even if we are aware of numerous other methods which are more sophisticated and surely more efficient for the only purpose of homologous relationship predictions (see for instance the reviews [21-24]).

The evaluation of the performances and impacts of the substitution matrices in homology search is a difficult task especially due to the lack of benchmarks or reference datasets, that is the knowledge of the true homologous relationships. Whereas such datasets may exist for model organisms, they lack crucially for many specific groups of species, such as Mollicutes. We first used a personal manually curated dataset of one-to-one orthologs. As the human expertise can not infer with certainty all evolutionary relationships between proteins of distant species, this set may contain errors and missing orthologs. These errors could bias our estimators of performance such as sensitivities and specificities. However, we can assume that these errors are independent of the compositional bias and will impact the results of both matrices the same way. Moreover, the initial set of orthologs which was further curated was obtained with classical predictions using BLOSUM62. Therefore, potential errors in the reference dataset will tend to favor the BLOSUM62 matrix rather than MOLLI60, and we may only underestimate the benefits of our new matrix. Indeed, if MOLLI60 did improve the ortholog predictions, the benefits were slight. This is due in part to the small size of the benchmark and also to the nature of the predictions: one-to-one orthologs are the easiest homology relationships to predict and the criterion of best hit reciprocity is very strong. The additionable value of MOLLI60 can only be shown for a small subset of the results and its impact is consequently minimized with respect to the large number of obvious one-to-one orthologs.

Concerning the homologous families analysis, to cope with the lack of "true" relationships, we used the phylogenetic profiles of the clusters as an evaluation criterion. We thus took full advantage of the large number of genomes at our disposal and their wide distribution in the evolutionary tree of Mollicutes. The computation of the core and pan genomes enables to have a first glance at the clustering properties in terms of biological considerations. The evaluation of the clustering is however based on two assumptions: that the orphan genes (genes without homolog) are rare and must be minimized and that the number of distinct clusters shared by all genomes must be maximized. These assumptions are guided by the parsimony principle. Indeed, in the last common ancestor of the considered species the core and pan genomes were equal and they diverged by gene gains and losses: the pan genome increased by gene gain and the core genome decreased by gene loss. Therefore a clustering resulting in a smaller pan genome and a larger core genome may invoke less gain and loss and be more parsimonious. It is however important to note that this hypothesis is strong and may not reflect the reality of Mollicute gene set evolution, that is the real evolutionary scenario may not be the most parsimonious. This is the reason why we performed a second evaluation based on the domain composition in and between families. This relies also on an hypothesis, that domain architectures are conserved inside the families and are specific to families.

Moreover, it concerns only a small portion of the genes, those for which the function is the best known and represented in studied organisms and this may bias the results towards more conserved genes. As the two evaluation methods relied on different assumptions and may have different biases, we argue that they complement each other and the fact that they gave the same trends strengthen our conclusion.

## Conclusions

In conclusion, we showed in this paper that we can improve orthology and homology predictions of Mollicute proteins by using a well-fitted substitution matrix, rather than the standard BLOSUM62. We argue that this result can be generalized to other groups of species with a marked compositional bias. The methodology we propose to construct such well-fitted matrices is very simple to carry out and could be useful in an increasing number of studies especially in the current context of massive growth of genomic data: the new sequencing technologies enable from now on to study specific groups of genomes with their specific and atypical nucleotide or amino acid compositions, notably for sequences coming from metagenomic studies.

## Methods

### Building of the matrix

To generate new matrices, we followed an approach similar to the one used for the well-known BLOSUM matrices [1]. This consists in learning amino acid frequencies and subsitutions from a reference set of aligned protein sequences. We used a set of 247 orthologous protein families among 14 public mycoplasma genomes (see the list in Additional file 4) for which the orthologous relationships were clearly ascertained.

These families were obtained by combining pairwise orthologous predictions (by BDBH) with high significance alignments, then selecting protein sets having at most one member per species. These families were further manually curated according to the protein annotations [25].

For each family, a multiple alignment was obtained using the program T-Coffee [13]. Then, using a custom perl script, we extracted from the alignments the contiguous blocks without gaps in any sequence and with length greater than a threshold (the minimum length was set to 15). And finally we applied the program BLOSUM [1] (retrieved from the following site ftp://ftp.ncbi.nih.gov/repository/blocks/unix/blosum/) on the whole set of blocks. This program clusters very similar sequences inside the blocks and counts all pairwise substitutions. The score of aligning the amino acid *a*_*i *_with *a*_*j *_is obtained with the following formula:

$$Sc\left(a_{i},a_{j}\right)=\frac{1}{\lambda }\log\frac{f\left(a_{i},a_{j}\right)}{f\left(a_{i}\right)\times f\left(a_{j}\right)}$$

where *f*(*a*_*i*_, *a*_*j*_) is the observed frequency of pairwise alignment of the two amino acids *a*_*i *_and *a*_*j*_, and *f*(*a*_*i*_) (resp. *f*(*a*_*j*_)) is the observed frequency of the amino acid *a*_*i*_. The level of clustering is determined with the parameter *c *which was set to 60. This means that sequences in a block with at least 60% identity are clustered together and will be considered as one sequence in the counting process. Then the matrix elements were normalized with the factor λ = 2 and rounded to the closest integer to obtain half-bit scores such as in the BLOSUM62 matrix.

For each matrix, relative entropy and expected values were obtained from the following formula:

$$\begin{array}{c} Entropy=\overset{20}{\underset{i=1}{\sum }}\overset{20}{\underset{j=1}{\sum }}f\left(a_{i},a_{j}\right)\times Sc\left(a_{i},a_{j}\right)\\ Expected=\overset{20}{\underset{i=1}{\sum }}\overset{20}{\underset{j=1}{\sum }}f\left(a_{i}\right)\times f\left(a_{j}\right)\times Sc\left(a_{i},a_{j}\right) \end{array}$$

The whole pipeline to build new matrices was implemented in perl scripts, which are provided in Additional file 2. It starts from a set of multi-fasta files (one for each protein family of the learning set) and outputs the final matrix in proper format to be used with alignment programs.

### Application of the matrix

#### Sequence data

All sequences and annotations of Mollicute proteins were retrieved from the Molligen database [11] (http://cbib1.cbib.u-bordeaux2.fr/molligen3b/). A complete list of genomes used in this paper is provided in Additional file 4.

#### Alignments and BDBH

Protein alignments were obtained with the program SSEARCH of the Fasta program package [18,26]. This program implements a fast version of the Smith and Waterman algorithm [27] and has the advantage of computing e-values without pre-computed statistical parameters. We also used the program BLAST [19] to compare the two strategies.

BDBH between pairs of genomes were obtained by aligning each protein sequence of one genome against all the protein sequences of the other genome and vice versa. A pair of proteins, *p*_1 _and *p*_2_, belonging respectively to genomes *G*_1 _and *G*_2_, was called a BDBH if *p*_1 _is the best hit of *p*_2 _among all proteins of *G*_1 _and reciprocally *p*_2 _is the best hit of *p*_1 _among all proteins of *G*_2_. The best hit was determined by the e-value (if not mentionned in the text) or the raw score of the pairwise alignment. BDBH relationships can be further filtered according to their alignment features (e.g. e-value) to discard less significant pairs.

#### Gap penalties

The substitution matrix is not the only parameter used to score gapped alignments. For each new matrix, it is thus important to optimise gap penalties. Indeed, the score of a gap has to be fixed relatively to the scores of matches and mismatches. Our approach to determine well-adapted gap penalties was empirical: we tried several combinations of gap open and gap extend penalties, and we evaluated them with respect to the performances in predicting orthologous relationships with the BDBH approach. We chose the gap penalties that gave the best compromise between sensitivity and specificity of ortholog predictions, using the ROC curves. We performed the same analysis for the BLOSUM62 matrix. For both matrices, we chose the following penalties: 10 for the opening of a gap and 1 for each extension of a gap. We verified that both matrices along with their gap penalties gave alignments of similar length.

#### Robustness evaluation

To evaluate if the obtained matrix is robust with respect to the set of protein families given as input, we used a Jacknife sampling approach. We generated 10 other matrices with the same procedure but with a smaller set of protein families sampled randomly from the original one. We generated two sets of samples: keeping either 90% or 50% of the initial protein families.

#### Reference set of orthologous relationships

The reference set of one-to-one orthologous relationships was obtained with standard BDBH predictions between 3 well annotated *mycoplasma *genomes: *Mycoplasma genitalium*, *Mycoplasma hominis *and *Ureaplasma parvum*. Each prediction was manually curated by biologists according to alignment features, functional annotations of the proteins and synteny analyses [25]. It contains 871 pairwise relationships among the 1634 protein coding genes of these three genomes.

This set enables to evaluate several methods of orthology predictions, by classifying the predictions as true positive, false positive (predictions absent from the reference set) and false negative (relationships of the reference set not predicted as orthologous); we denote by TP, FP and FN the amount of predictions in each category respectively. Then we can compute sensitivity and specificity values for each method, such as $\text{sensitivity}=\frac{TP}{TP+FN}$ (also called the true positive rate) and $\text{specificity}=1-\frac{FP}{TN+FN}$ or 1 - false positive rate (TN being the number of true negatives, e.g. relationships absent from the reference set and from the predictions). ROC curves were built by plotting the sensitivity versus the specificity for each cut-off of the evaluation criterion (alignment score or evalue). Differences between ROC curves were tested by comparing their area under the curve using the R package pROC [28].

The variability of alignment evalues was analyzed with respect to the matrix and the status of the prediction (either TP or FP), using an Analysis of Variance. To test the effect of each factor and in particular if the discrimmination between TP and FP evalues is different according to the matrix used (interaction factor), we used the following model:

$$log10\left(evalue_{i}\right)=\mu +a*MOLLI60_{i}+b*TP_{i}+c*MOLLI60_{i}*TP_{i}+e_{i}$$

with *MOLLI*60 and *TP *being boolean variables indicating respectively whether the prediction has been obtained with MOLLI60 and whether it is a true positive. *e *is the error and is assumed to be normally distributed. The estimation of the parameters of the fitted model were the following:

μ=-8.4,a=-0.7,b=-25.9,c=-19.5.

#### Clustering of protein families

We built homologous protein families between 24 Mollicute genomes with a simple clustering method: first we runned all-vs-all protein alignments with SSEARCH using one of the two matrices. Then, we grouped together in a same cluster any two proteins with a significant alignment, that is with an e-value less than a given threshold and covering at least 50 percent of the longest protein. In other words, we built a similarity graph with each node being a protein and each edge connecting two proteins with a significant alignment. The clusters were then the connected components of this graph, that is any two custers are completely disjoint in this graph (also known as single-linkage clustering).

Domain architectures were attributed to clusters containing at least one sequence with at least one predicted domain. We considered the domains predicted by running InterProScan on each proteic sequence against the Pfam-A database of domains [29]. The domain architecture of a protein was defined as the set of domain families predicted on the protein, as identified by their clan_id.

## Authors' contributions

CL implemented the method and performed the analyses. CL, AB, PT and PSP designed and conceived the method and experiments. AB and PSP built and manually curated the reference datasets. CL and PT wrote the manuscript. PSP, CT, FTa and FTh wrote the grant application upon which this project was built. All authors read and approved the final manuscript.

## Supplementary Material

Additional file 1 **The MOLLI60 matrix**. Click here for file

Additional file 2 **perl scripts for building new matrices**. Click here for file

Additional file 3 **Supplementary figures**. Click here for file

Additional file 4 **List of genomes used in these analyses**. Click here for file
