# Supplementary material for: A novel substitution matrix fitted to the compositional bias in Mollicutes improves the prediction of homologous relationships
Source: BMC Bioinformatics. 2011 Nov 24;12:457. doi: 10.1186/1471-2105-12-457 (PMC3248887; doi:10.1186/1471-2105-12-457)
Supplement: Additional file 3 — Supplementary figures. [file 1471-2105-12-457-S3.PDF]

Additional File 3 for

A novel substitution matrix fitted to the compositional bias in Mollicutes improves the prediction of homologous relationships

Claire Lemaitre [*,1,2], Aurélien Barré[1], Christine Citti[3,4], Florence Tardy[5], François Thiaucourt[6], Pascal Sirand-Pugnet[7,8] and Patricia Thébault[*,1,9]

[1] *Université de Bordeaux, Centre de Bioinformatique et Génomique Fonctionnelle Bordeaux, F-33000 Bordeaux, France*
[2] *Equipe SYMBIOSE, INRIA Rennes Bretagne Atlantique, Campus de Beaulieu, F-35042 Rennes, France*
[3] *Université de Toulouse, ENVT, UMR 1225, F-31076 Toulouse, France*
[4] *INRA, UMR 1225, F-31076 Toulouse, France*
[5] *Anses, Lyon Laboratory, UMR Mycoplasmoses of Ruminants, 31 Avenue Tony Garnier F-69364 Lyon cedex 07, France*
[6] *CIRAD, UMR CMAEE, Campus de Baillarguet, F-34398 Montpellier, France*
[7] *Université de Bordeaux, UMR 1332, 71, avenue Edouard Bourlaux, F-33140 Villenave d'Ornon, France*
[8] *INRA, UMR 1332, 71, avenue Edouard Bourlaux, F-33140 Villenave d'Ornon, France*
[9] *Université de Bordeaux, Laboratoire Bordelais de Recherche en Informatique, UMR 5800, F-33405 Talence, France*
[*] *Corresponding authors : CL: claire.lemaitre@inria.fr, PT: patricia.thebault@u-bordeaux2.fr*

Figure S1 - Comparison of SSEARCH and Blast.

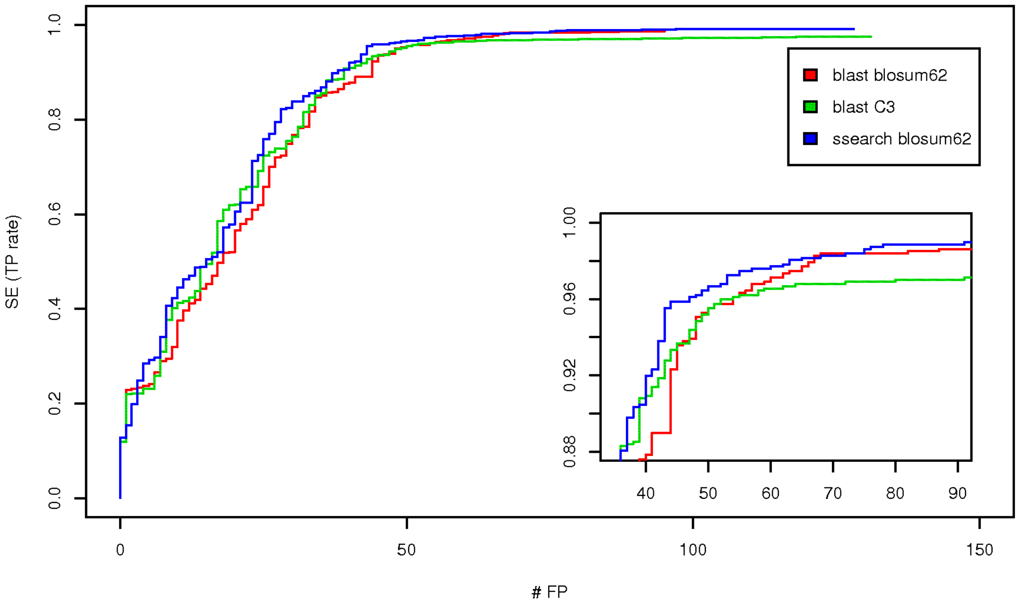

Figure S1: ROC curves of one-to-one orthologous relationship predictions using the Bi-directional Best Hit method with two alignment programs : SSEARCH and BLAST. Both programs used the BLOSUM62 matrix. BLAST was used with (C3) or without the option -C3 which takes into account the compositional bias.

Figure S2 - Evaluation of the clusterings with domain architecture metrics

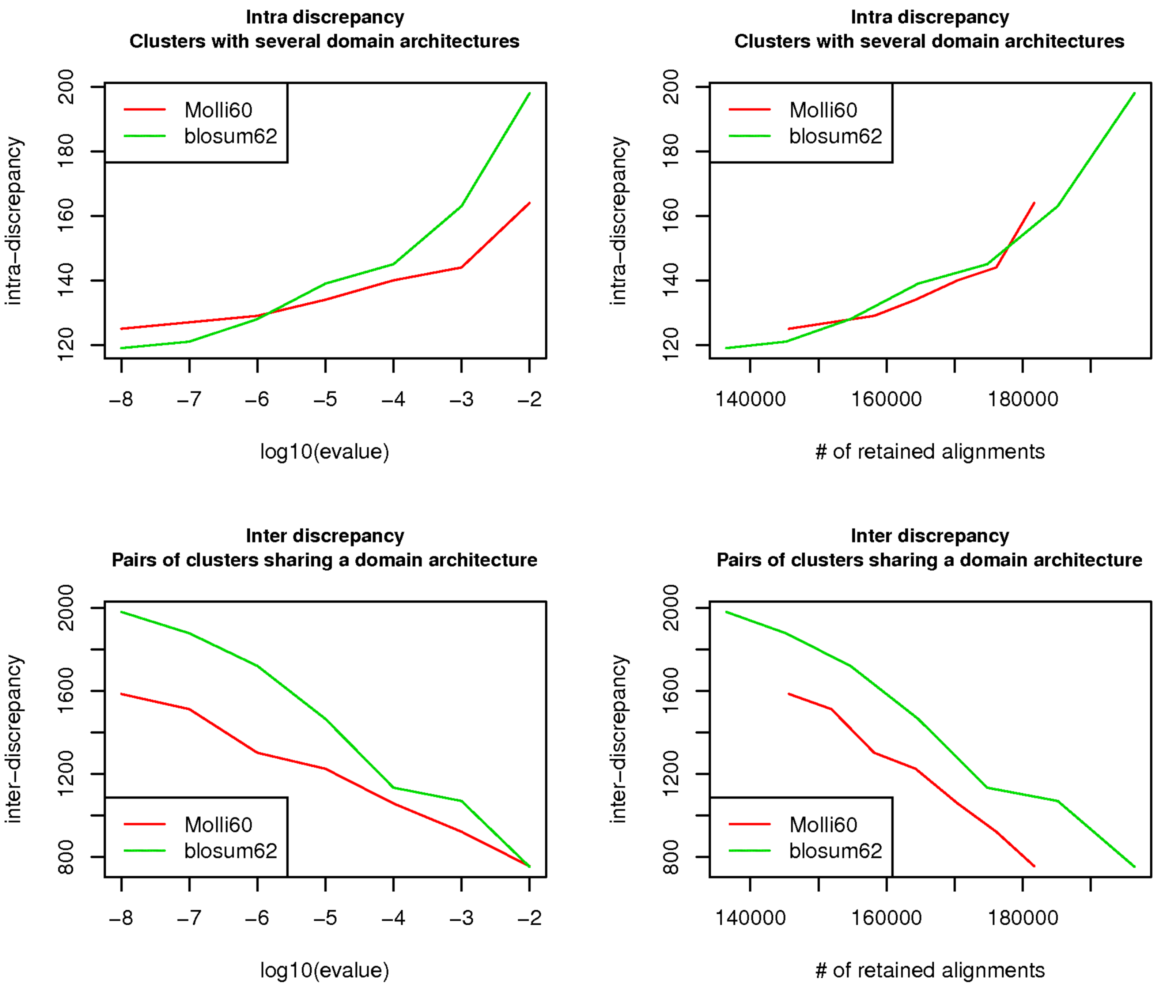

Figure S2: Comparison of the clusterings obtained with the two matrices MOLLI60 (in red) versus BLOSUM62 (in green), using two domain composition metrics as a function of the e-value threshold (left) or the number of retained alignments (right). The first metric $intra - discrepancy$ (top) counts the number of clusters with at least two distinct domain architectures among its protein members, it is weighted by the number of different domain architectures represented in the cluster. The second metric $inter - discrepancy$ (bottom) counts the number of pairs of clusters having in common at least one domain architecture among its protein members. These metrics evaluate the homogeneity in terms of domain composition of the clusterings. A better clustering will have both metrics the lowest.
